# Blockchain Technology, Cryptocurrency: Entropy-Based Perspective

**DOI:** 10.3390/e24040557

**Published:** 2022-04-15

**Authors:** Feng Liu, Hao-Yang Fan, Jia-Yin Qi

**Affiliations:** 1 School of Computer Science and Technology, East China Normal University, Shanghai 200062, China; 2 Institute of Artificial Intelligence and Change Management, Shanghai University of International Business and Economics, Shanghai 201620, China; 3 School of Statistics and Information, Shanghai University of International Business and Economics, Shanghai 201620, China; 21349074@suibe.edu.cn

**Keywords:** blockchain technology, cryptocurrency, Kolmogorov entropy, DAO, metaverse

## Abstract

The large-scale application of blockchain technology is an expected to be an inevitable trend. This study revolves around published papers and articles related to blockchain technology, relevance analysis and sorting through the retrieved documents with six core layers of blockchain: Application Layer, Contract Layer, Actuator Layer, Consensus Layer, Network Layer and Data Layer. Based on the analysis results, this study found that China’s research is more towards the preference and application of landing and industry and smart cities with blockchain as the underlying technology. International research is more focused on the research of finance as the underlying technology of blockchain and tries to combine crypto assets with real industries, such as crypted assets and payment systems for traditional industries. This paper studies the impact of monetary entropy on cryptocurrencies in smart cities and uses the monetary entropy formula to measure the crypto-economic entropy. We use Kolmogorov entropy to describe the degree of chaos in the cryptocurrency market in a smart city. The study illustrates the current status of blockchain technology and applications from the perspective of cryptocurrency in a smart city. We find that smart cities and cryptocurrencies have a mutually reinforcing effect.

## 1. Introduction

Since Nakamoto published ‘Bitcoin: A peer-to-peer electronic cash system’ in 2008, the development of Bitcoin has been up and down, but its underlying blockchain technology has received more and more attention in recent years. In 2019, China decided to take blockchain as an important breakthrough for independent innovation of core technologies, and to accelerate the development of blockchain technology and industrial innovation in smart cities with blockchain technology. The main application scenarios could be found in areas like people’s livelihood services, urban governance, industrial economy, and ecological livability. The most eye-catching international use of blockchain technology is the rapid development of cryptocurrencies. This study will analyze the development direction of cryptocurrency from the perspective of world political economy, Bitcoin futures ETF, NFT, DAO, Web3.0. At the same time, along with the rising inflation in the United States and the impact of the COVID-19, the American public’s attention to encrypted assets has increased and global encrypted assets have reached the level of trillions of dollars. The international application of blockchain is more focused on the financial field and focused on the virtualization industry. This study uses two scenarios of smart city and cryptocurrency to correspond to China’s and other countries’ concerns, respectively, to describe the development of blockchain technology in China and other countries, and they can get useful inspiration by comparing different development paths to promote the development of industry and blockchain technology. This paper uses entropy to analyze the performance of cryptocurrency. The cryptocurrency market and smart city represent a chaotic state, and we introduce Kolmogorov entropy to measure the degree of chaos, which is the direction of our future research. The study is a qualitative study, and we will do more quantitative studies in the future to measure specific monetary entropy, crypto-economic entropy, urban entropy, Kolmogorov entropy etc.

The rest of this article is: Section 2 Entropy in cryptocurrency markets; Section 3 Study of blockchain technology; Section 4 Solutions about blockchain technology; Section 5 Cryptocurrency and entropy; Section 6 Discussion; Finally, Section 7 Concludes this work.

## 2. Entropy in Cryptocurrency Markets

Thermodynamics-related theories in physics have driven the development of economics in the past 100 years, especially when the theory of ‘entropy’ in thermodynamics emerged, which greatly promoted the development of modern economic theories. For example, entropic economics, complex economics, and quantum economics, etc. This shows that ‘entropy’ has bridged the gap between economics and physics and has had a dramatic impact on mainstream economic theory. The concept of ‘entropy’ originated in the 19th century, and first indicated that part of the energy of a steam engine could not be transformed into useful work due to friction and other reasons, and ‘entropy’ measured the missing energy in this part. The first mathematical definition of ‘entropy’ is shown below [1].

(1) $$C=\frac{1}{T}q$$

(2) $$\Delta c=\left(\frac{1}{T_{2}}-\frac{1}{T_{1}}\right)q$$

where Δ*c* represents the change in entropy and *q* is the heat transferred from an object with temperature *T*_1_ to other object with temperature *T*_2_. A slightly different definition of entropy, being a measure of the molecular disorder of the system, was formulated by Boltzmann. It has the following form:

(3) $$C=K\ln\left(m\right)$$

where *K* is the Boltzmann constant, while *m* is the number of microscopic states.

‘Entropy’ in thermodynamics can be used in the social sciences as a general measure of the disorder in a system. For example, the concept of ‘corporate entropy’ is used in management and organizational sciences, and should be understood as a loss of productive energy. The entropy in an organization is always growing, just like the thermodynamic entropy in the universe. As the concept of ‘entropy’ continues to evolve, the various definitions of entropy can be made more specific and applied to specific financial scenarios. John Bryant in his book provides a careful mathematical description of ‘entropy’ in the economy with the following expression shown in, it has the following form:

(4) $$c=\ln\left(\frac{v}{L}\right)$$

where *v* represents the volume of economic activity and *L* represents the constrained level of that activity. The change in the entropy of the economy per unit of time can be expressed in a more precise formula.

(5) $$dC=\left(w-wn+1\right)\frac{dv}{v}=\left(1+\frac{1-n}{r}\right)\frac{dv}{v}$$

where *dv/v* represents the growth rate of volume flow, *w* is the lifetime factor, n represents the elasticity index, and r is the natural rate of return. The factor (*w − wn + 1*) is called the marginal entropy index, and the integration of Equation (5) yields the entropy generation per unit time using the following mathematical form:

(6) $$c=\left(w-wn+1\right)\ln\left(v\right)+c_{0}=\left(1+\frac{1-n}{r}\right)\ln\left(v\right)+c_{0}$$

Equation (6) can be used to describe the monetary entropy, in which case the rate of return approximates the long-run average or natural level of the velocity of money circulation.

Kolmogorov entropy is an important quantity to characterize chaotic systems. In different types of dynamical systems, the value of *K* is different, and in systems with chaotic motion, the value of *K* is greater than zero. the larger the value of *K*, then the greater the rate of loss of information. the formula for Kolmogorov entropy is shown below [2].

(7) $$k=-\underset{\tau \to 0}{\lim}\underset{\varepsilon \to 0}{\lim}\underset{d\to \infty }{\lim}\frac{1}{d\tau }\sum_{i_{1},\ldots ,i_{d}}^{}p(i_{1},\ldots ,i_{d})\ln p(i_{1},\ldots ,i_{d})$$

where *p*(*i_1_, …, i_d_*) is the joint probability and ε and d are fixed values. Equation (7) can portray the degree of chaos of cryptocurrency and smart cities. Samet Gunay andand Kerem [3] showed that cryptocurrency markets are not random but chaotic. In the present, this means that the short-term prediction of the cryptocurrency market may be achievable, but it is completely unpredictable in the long-term prediction.

R. Fistola and R. A. La Rocca [4] studied the measurement of urban entropy, which is a complex system, and divided the urban system into five subsystems, each of which contains several influences that are used to measure the entropy of the city. In addition, it is beneficial to keep the entropy of a city within a reasonable range, but when the entropy is too low or too high, this can lead to a ‘fragile’ city or reduce the ability to sustain development. Dehouche [5] uses an approximate entropy approach to verify the reasons for the exponential and persistent fluctuations of the bitcoin price, using data such as the daily price of bitcoin, the price of gold, and the SandP 500 index, and calculating their standard deviations. Pele, DT and Marinescu-Pele [6] used the entropy of bitcoin’s daily returns to predict the daily value-at-risk of bitcoin and demonstrated that entropy outperforms the classical GARCH model, and the following conclusions are drawn: There is a strong positive correlation between the daily log price of bitcoin and the intra-day return entropy, indicating that entropy has predictive power for bitcoin price. Grilli and Domenico [7] introduced the concept of Boltzmann entropy into cryptographic digital currencies and used Boltzmann entropy to predict the price change trend of cryptographic digital currencies.

The entropy of the cryptocurrency market and the traditional currency market will be affected by various factors, as shown in Table 1 such as inflation rate, fiscal deficit level, interest rate volatility, and high cost of currency management in the traditional currency market. For the cryptocurrency market, the basic blockchain peer-to-peer and decentralized technology of cryptocurrency saves a lot of operation and management costs, meanwhile, along with the emergence of NFT, Dao, Web 3.0, metaverse in the cryptocurrency market, they are constantly optimizing the ecological environment of the crypto market.

In economic reform, ‘entropy’ can be used as a tool for future monetary reform, in this paper mentioned cryptocurrency and smart city, in which includes a large number of ‘entropy’, such as: ‘monetary entropy ‘, ‘education entropy’, ‘transportation entropy’, ‘ecological entropy’, etc., when cryptocurrencies appear in the monetary market as well as transforming the urban governance model to ‘smart city’ mode is transformed, it is in reducing the degree of chaos within the original system, reducing the lost part of the system operation, improving the operation efficiency, and then introducing ‘entropy’ into the currency market and city construction for the global crypto market and city governance model.

In this paper, we introduce the concept of Kolmogorov entropy to smart cities and cryptocurrency, and use Kolmogorov entropy to measure the degree of disorder in the monetary market of smart cities as a way to speculate whether the smart cities are developing stably.

## 3. Study of Blockchain Technology

This study on blockchain technology focuses on the six layers of the blockchain, which are: Application Layer, Contract Layer, the Actuator Layer, Consensus Layer, Network Layer and Data Layer. Detailed technical research of studies is shown in Table 2.

For the Application Level, the China’s studies focus on social industries such as e-commerce, education, taxation, medical care, intellectual property and social governance. This paper [78] believes that the introduction of blockchain, edge computing and other technologies under the distributed architecture computing network can provide digital power for smart earth applications. International studies focus more on the economic and financial fields such as investment, payment, identification, anti-money laundering, business process re-engineering, finance, etc. For the Contract Layer, the Chinese studies focus on tying up the industrial chain through the contract state machine, to implement its designed smart contract into the industry. This paper [79] studies a new type of decentralized threshold signature protocol. By combining distributed the key generation protocol and BLS signature, a set of threshold signature protocol with fixed signature length that can be participated by multiple parties is designed. The international studies focus more on the blockchain smart contract itself, through the design of the contract layer protocol to serve the blockchain network. For the Actuator Layer, both Chinese and international studies focus on the design of reputation-based game model. In terms of preferences for technical scenarios’ landing. This paper [80] proposes a two-party elliptic curve digital signature algorithm suitable for blockchain. Through the mathematical logic of the given signature algorithm and its security model, it is integrated into the blockchain for evaluation, which proves the feasibility of the scheme. Chinese studies focus on the integration of actuation and data sharing scenarios, while international studies focus on the blockchain network improvements. For the Consensus Layer, both Chinese and international studies prefer the improved consensus algorithm for existing PoW, (D) PoS and PBFT. Few new consensus algorithms have been proposed. For the Network Layer, China preferences is for efficiency improvements through external software and services, while the international preference is more focused on technical improvements to the blockchain network layer. For the Data Layer, Chinese preference is to apply data in the security field, while the international focus is more on research and improvements of the blockchain data layer, such as data structure, data expansion and chain structure improvement. For example, in the case of ensuring data security and credibility with the help of the blockchain double-chain structure, the endogeneity is improved [81]. The problem of low efficiency of data interaction between middle and platform, etc.

To sum up, based on the six levels of analysis mentioned above, we can clearly see the similarities and differences between Chinese and foreign research directions: At the Application Level, China’s attention is paid more to the application of actual scenarios, while international attention is paid to the application in the financial field; at the Contract Layer, China’s focus on the application of smart contracts in the industrial chain, while the international research on the contract itself has been strengthened; in Actuator Layer, both Chinese and international studies focus on the design of game model; in Consensus Layer, both are researching How to improve the consensus mechanism; in Network Layer, China attaches great importance to external software and services, while the international community pays more attention to improving the blockchain network itself; In Data Layer, domestic data is used more in the field of security, while the international community pays more attention to the research of the data layer itself.

## 4. Solutions about Blockchain Technology

Based on the research differentiation in China and internationally, we can condense the following table conclusions on the six-layer structure of the blockchain, as shown in Table 3.

By analyzing the domestic and international research of blockchain, we can make relevant references. Considering that Chinese blockchain research is more linked to the industrial chain and rooted in the real economy applications. In terms of the core algorithms, we can learn more from international research of improvements of the contract, consensus and network layers. We can also take full use of China’s achievements in the security and privacy design. Thus, by combining the advantages of both to achieve and implement the whole.

## 5. Cryptocurrency and Entropy

**Figure 1:**
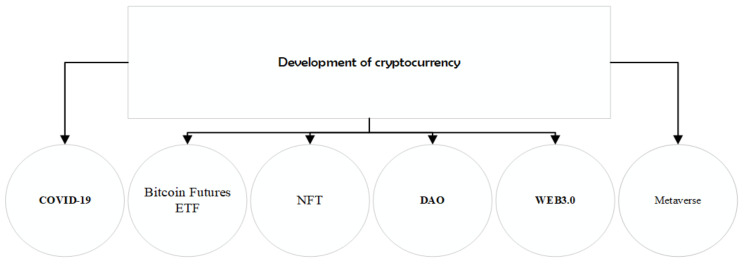
Development of cryptocurrency.

Cryptocurrency is one of the most important scenarios for the application of blockchain in the smart city, and with the development of DEFI and cryptocurrency, which the relationship will become closer and closer, and cryptocurrency may play an important role in the international financial market of the smart city. As shown in Figure 1, which analyses the factors that affect the development of cryptocurrencies.

### 5.1. The Global Epidemic and Inflation Will Accelerate People’s Interest in Encrypted Assets

The COVID-19 epidemic in 2020 and the recession that came with it hit individuals, small businesses and governments in a short period of time, but the consequences of this are also complex, providing opportunities for innovation on the one hand, causing social unrest and economic hardship on the other hand. In terms of monetary policy, the Federal Reserve is moving constantly and its balance sheet is expanding at a very fast pace. The Fed’s fiscal deficit reached $3.1 trillion in 2020 and is expected to reach $3 trillion in 2021, creating insecure expectations for the population. The rapid changes in society as a whole during this period forced investors to react accordingly and new investment trends are developing. Based on this, our survey shows a significant increase in interest in safe-haven assets compared to the results of 2019, and among the safe-haven assets are cryptocurrencies such as Bitcoin, a phenomenon that is within the forecast range. During the COVID-19 epidemic in 2020, which set back the entire traditional financial market. Cryptocurrencies played a role as a hedge against risk, with Bitcoin being ideal for risk averse individuals in the face of downward pressure in financial markets [82]. Further studies found that bitcoin in both domestic and cross-border entities served to achieve diversification benefits and risk mitigation, acting as a ‘safe haven’ [83,84,85]. The cryptocurrency market shows higher levels of cross-correlations with the others during the COVID-19 periods, in which it is strongly cross-correlated itself [86]. From these results, it can be concluded that the COVID-19 promoted bitcoin investments, 63% of the bitcoin investors were influenced by the COVID-19 epidemic in the past year, and it has boosted the price of bitcoin [87].

Based on the analysis of the socio-political and economic status quo, the traditional financial market is vulnerable to severe shocks, and the market is unstable. The Fed accelerates money printing and inflation, and the issue of asset preservation has received more and more attention. Cryptocurrency has quickly attracted attention due to its excellent properties such as security, limitedness, and easy liquidity. Once investors make profits in the encrypted market, they will attract more investment. This is based on the external unstable financial environment make cryptocurrency investment market boom.

### 5.2. Bitcoin Futures ETF

Several Bitcoin ETF applications have been rejected since 2013, citing market manipulation, fraud and failure to protect consumers. On 19 October 2021 at 9:30 am EST, the SEC approved the issuance of a bitcoin futures ETF, which will probably lower the investment threshold for bitcoin after the adoption of the bitcoin futures ETF. With the caveat that the purchase of a bitcoin futures ETF is an indirect investment in bitcoin, not a direct investment. The purchase here is simply a futures contract, which is less effective at tracking the price of bitcoin. For better tracking of the price of bitcoin, a spot ETF would be a better option, but is not available at this time as the liquidity of bitcoin may not be sufficient for the liquidity required by institutions. Bitcoin futures ETFs are regulated to track the spot bitcoin price using CME regulated futures contracts, which can be regulated by traditional exchanges for added security. However, there are two disadvantages to bitcoin futures ETFs: one is that futures have a premium and discount problem that if the bitcoin price changes by 1% in an hour, the bitcoin futures ETF may drop by 2%, which is a significant deviation, meaning that futures ETFs do not track the bitcoin price well enough; the other is that investment costs are high, as futures contracts have an expiry date, and near the expiry date. This is the cost of shifting the futures ETFs, which can eat up some of the profits, so bitcoin futures ETFs are not suitable for long-term holdings, and can be used for short-term investments. In reality, Bitcoin also requires the retention of an associated Bitcoin wallet as well as registration with a cryptocurrency exchange, which remains unknown to those unfamiliar with the space and requires a degree of self-education. With a Bitcoin futures ETF, investors do not need to worry about private keys, storage or security. They own shares in the ETF, just like their shares, and have access to the cryptocurrency market without having to buy and hold cryptocurrency. Bitcoin futures ETFs are managed by companies that buy and hold actual bitcoins, with prices linked to the bitcoins held in the fund. The company lists the ETF on a traditional stock exchange and we, as investors, can trade the ETF just like any other stock.

In conclusion, for investors who are reluctant to invest in bitcoin directly due to price, security and regulation. buying a bitcoin futures ETF is an opportunity to invest in cryptocurrencies, which adds to the focus on cryptocurrencies in the traditional financial markets, which is why I believe that bitcoin futures ETFs are an important option for some investors in the future.

### 5.3. NFT

**Figure 2:**
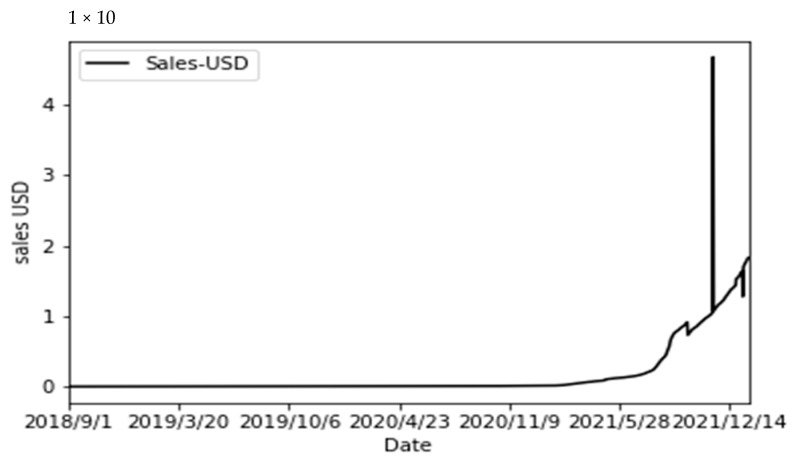
Total NFT transaction volume.

The NFT market has been very popular in the past two or three years. The concept of NFT originated from Ethereum. NFT is a form of cryptocurrency [88]. NFT can also be regarded as the ownership certification for identifying virtual assets. They can be largely subdivided into several small parts. NFT is a subdivision that cannot be carried out. Such tokens can be bound with virtual or digital assets to form their unique identifiers. After forming NFTs, they can be freely traded in the market, which greatly promotes the development of virtual currencies and stimulates the prosperity of the DAPP market. By using NFT on smart contracts, creators can easily prove ownership including ‘picture’, ‘video’, and ‘artwork’, so that intellectual property rights can be properly protected. NFT no matter the transaction many times, its original creators have drawn royalties, which has also spurred the development of digital artworks in disguise. The real-time data as of the time when the author wrote the paper shows that the current total transaction value of NFT are close to 16.4 billion US dollars as shown in Figure 2 (Data from: nonfungible.com (accessed on 30 December 2021), and its growing market and large returns have attracted people’s attention.

**Figure 3:**
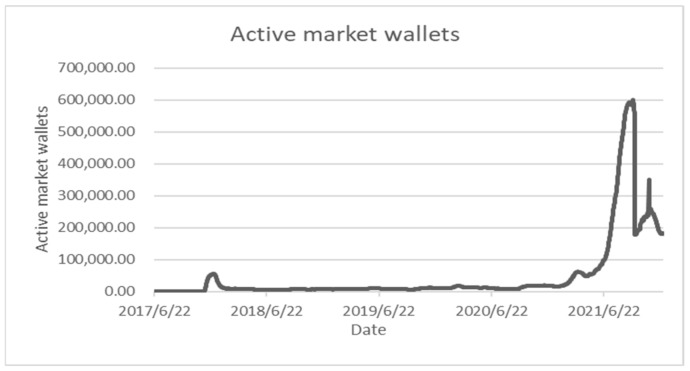
The number of active wallets related to investing in NFTs.

In the past 24 h, the transaction volume of NFT was US$49,858,590.46 [89], and the transaction volume of cryptocurrency was US$102,099,049,616 [90]. The transaction volume of NFT accounted for about 0.05% of the transaction volume of cryptocurrency. It can be seen that NFT is still developing at the initial stage of 2020, but there is a significant increase compared to the same period in 2020, and its growth can also be seen from other indicators. In April 2020, the number of initial transactions was 25,729, the number of secondary transactions was 8589, and the current number of initial transactions is 14,852,246 times, and the number of secondary transactions was 13,359,460 times. In addition, the active level of investment can also be seen from the number of active wallets. As shown in Figure 3 (Data from: nonfungible.com (30 December 2021), the number of wallets investing in NFT-related products in the past three months has increased from about 97,000 to about 200,000 now, which also reflects given the latest investment trends in cryptocurrencies, NFT-related products will continue to attract a large influx of cryptocurrencies.

In addition, the concept of the ‘metaverse’ rises in 2021, and 2021 is also known as the first year of the ‘metaverse’. With the renaming of Facebook and the listing of the Roblox game company, the concept of the Metaverse instantly aroused thousands of waves, leading to the influx of other Internet giants, which also led to the accelerated development of the industry and the entry of capital, and it would quickly integrate human resources. This further stimulated the market to have a high valuation for the Metaverse, and finally formed a market outlet industry. This way, investment and speculation in the market will come one after another. Now the total value of the Metaverse-related industries has reached about 714 million USD. In the future, running the metaverse will need to include a large number of NFTs, cryptocurrencies and NFT-related products will be used in the future virtual world. It can be used, leased and bought and sold in the metaverse, which requires a large amount of cryptocurrencies to promote the market’s investment interest in cryptocurrencies.

In a word, NFT will enhance the development of the entire encrypted market [91], such as: existing encrypted games; one can continue to buy and sell NFT products in the game; protect digital collectibles, protect digital assets and intellectual property rights; finally, prosper the entire metaverse. From the current point of view, NFT-related products are similar to the process of re-enclosure. Redistributing resources and wealth will attract a large number of people to participate, which also requires the use of cryptocurrencies, and it will be one of the investment directions of cryptocurrencies in the future.

### 5.4. DAO

DAO is a decentralized autonomous organization. The decision of the organization is made by the group. The organization will also issue its own tokens (but not necessarily every organization will issue it), some of tokens held or those participating in the DAO. The project, which determines the size of the voting power, DAO can be a company where everyone is the boss, both consumers and owners, which actually shortens the distance between the market and the organization, and through DAO efficient voting, decision-making. It can shorten the mechanism of decision-making and execution, thereby improving the efficiency of the organization. The business model of DAO can be very promising in the future. NFT is used to illustrate that the current NFT market has a phenomenon of oversupply and lack of development potential. If DAO appears at this time, its development can be promoted. For example, Party-DAO, which focuses on investing in NFT projects, its goal is to gather the power of retail investors to obtain funds through crowdfunding to buy NFTs. After the purchase is successful, the internal tokens will be distributed proportionally to record the NFT purchase share. If you want to sell NFTs, you need to initiate a vote within the organization. If the support exceeds 50%, a public auction will be held, and the proceeds will be divided equally according to the proportion. This not only promotes the trading of NFTs, but also promotes more cash flow into the cryptocurrency market, so DAO is a “good medicine” to improve the entire decentralized finance.

As shown in Table 4 DAO companies are divided into eight categories according to their functions and business. DAO-operating systems: The main task is to help users create DAO; Investments DAOs: To raise funds to invest in projects recognized by DAO internal members, if there is profit, it will be divided proportionally, otherwise the risk will be shared; Grants DAO: If you want to improve the internal DAO, you need to initiate a proposal as a member. If the proposal is passed, you can get a bonus to complete your own ideas; Collector DAOs: It belongs to the collection DAO, that is, only invests in NFT projects, and the profits are divided proportionally, otherwise you need to share the risk; protocol DAOs: Mainly do cryptocurrency lending business on the blockchain; Service DAOs: Provide services to DAO, such as fundraising to buy NFT, or analyze data; Social DAOs: Mainly do online discussions and interactions; Media DAOs: Blockchain news.

If you want to join any of the above organizations, you need to hold some of the cryptocurrencies specified by the organization or participate in the project together. These are two main ways to join the organization. No matter which method is used, it is inseparable from cryptocurrencies. It is also an important choice in investing in cryptocurrencies.

### 5.5. Web 3.0

Web3.0 is a newly proposed technical means; it is based on the blockchain technology to develop the next generation of a brand-new and efficient network world. In the era of web 1.0, that is, the early Internet, you could only browse information related to web pages; In the era of web 2.0, mobile phone terminals appeared, allowing users to interact with the platform a lot. Absolute management and supervision rights; In the future web3.0 era, there is no central agency for review, smart contracts are cornerstone of web3.0 operation, users have absolute control over the privacy of data, and users can combine their own actual situation. Data are sold for profit. Web3.0 is not a direct invest cryptocurrency; it is more like participating in the maintenance of the network together. As a part of it, the cryptocurrency reward obtained by contributing one’s own strength, which will mobilize the power of global netizens to jointly the future network world created. Although cryptocurrency investment is not directly reflected here, as long as you participate in it, you can get the corresponding governance token rewards, which is another way to invest.

For example: ‘brave’ browser, which now has 42 million users worldwide, is based on the concept of web3.0, and its focus on privacy protection, which will not be interfered by any advertisements. One can also choose to watch advertisements, you will be rewarded with bat tokens. This can form a win-win situation for users, advertisers and the platform.

The emergence of Web3.0 will be of significant epoch-making, it will change the operation mode of the entire encrypted economy and the development and profit model of the entire Internet company. Based on blockchain technology in the era of web3.0, every user’s attention has been mobilized. This way of operation will also be more private, secure, and efficient.

### 5.6. Metaverse

In the science fiction “*Snow Crash*“ published in the 1990s, a virtual world was constructed, which is a concept of “metaverse” in today’s view. In 2021, the concept of “metaverse” will rise. Metaverse’ first year. There are many definitions of the metaverse now, it is necessary to build a virtual world with a strong sense of experience. The future Metaverse will not disappear with the collapse of an Internet company, and the economic system in the metaverse will not disappear. To be interconnected with the economic system of the real world, more and more internet giants are optimistic about the development potential of the Metaverse. With the renaming of Facebook and the listing of the Roblox game company, the concept of the Metaverse instantly aroused thousands of waves. Leading to other Internet Giants are also pouring to speed up the layout of the Metaverse, which will lead to the accelerated development of the industry and the entry of capital, and it will further accelerate the integration of manpower and resources, which will further enhance the market’s value expectations for the Metaverse, and then form a market outlet industry. In this way, investment and speculation in the market will continue to flow, and the total value of the industry related to the Metaverse has now reached about 714 million US dollars. In the future, running the metaverse will probably need to include a large number of NFTs, cryptocurrencies and NFT-related products will be used in the future virtual world. It can be used, leased and bought and sold in the metaverse, which requires the use of a large number of cryptocurrencies to promote the market’s investment interest in cryptocurrencies.

Since many companies do not currently have data related to each other, the entire data will be integrated in the future metaverse. This step may become a revolution in the “interface” between the real world and the digital world. Since the current metaverse is still in the early stage of development, there will be huge changes in the future. Metaverse-related projects are also being explored in the industry. At present, NVIDIA has created the NVIDIA Omniverse platform, which can use digital twin technology to build a virtual factory in a virtual world, and real products can be tested. Its data can be Synchronized with the real factory, which can liberate productivity, reduce costs and increase efficiency.

The metaverse must bring about drastic changes, which require continuous innovation of a large number of technical means. At the same time, virtual currency is booming, which also promotes the development of the virtual world. However, since the construction of the metaverse and the entire virtual world is still in its infancy, it should be subject to the supervision of relevant international organizations. It is possible to explore the use of legal digital currency as the transaction currency of the metaverse.

Based on the monetary entropy formula, this study gives the concise measurement Equation (8). The six influencing factors, such as inflation, Bitcoin futures ETF, NFT, DAO, Web 3.0 and Metaverse, are measured separately in Equation (8). *Ce_i_* represents the entropy value of the 6 influencing factors, *w* is the lifetime coefficient, *N* represents the elastic index, *V* stands for the volume of economic activity, *C_o_* is a constant.

(8) $$Ce_{i}=(W_{i}-W_{i}N_{i})\ln(V_{i})+C_{o}$$

Equation (9), *Ce* represents the measurement of the overall development of crypto-economy in smart cities under the combined effect of six factors such as NFT, DAO, WEB3.0, etc. After we get the crypto-economy entropy, we can infer the financial market operation in the smart city.

(9) $$Ce=C(Ce_{1}\ldots Ce_{n})$$

After analyzing the changes in the world’s political economy, NFT, DAO, WEB3.0, bitcoin futures ETF, Metaverse, it can be seen the entire traditional financial market is changing, online world is also undergoing restructuring. At the same time, with the development of communication technology, it will reshape a new economy patterns and social formations. Blockchain technology enables everyone to have the opportunity to integrate into economic and social development. However, because of it is still in the early stage of development and the development rules are not perfect, it is necessary to improve supervision capabilities to meet the challenges of the ever-changing encrypted economy in the future of smart city.

## 6. Discussion

In this paper, we introduce a smart city as a scenario and use Kolmogorov entropy to calculate the degree of chaos of cryptocurrency and smart city. Smart cities applying cryptocurrency can introduce monetary entropy, which may be one of the future research directions. In addition, smart cities can also use entropy to measure the development of the whole urban system. The following are the main development directions of smart cities.

**Figure 4:**
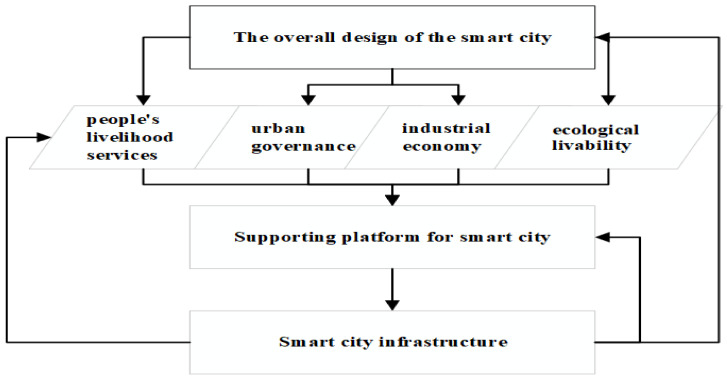
Smart city construction based on blockchain technology.

The construction of smart cities is the development direction of major cities in China in these years, along with the rapid development of information and communication technology (ICT) such as artificial intelligence, 5G, blockchain and Internet of Things, which make the technical means of building smart cities more and more perfect, but at the same time, cities generate a large amount of data and huge data transmission tasks, which leads to the problem of information security, and blockchain technology can rebuild trust in society and solve the sharing problem. However, the application of blockchain technology in smart cities is a difficult system project, requiring the construction of a secure and credible smart city data infrastructure, a city-level multi-level blockchain public service platform, and a breakthrough with a focus on government service innovation to promote the gradual implementation of blockchain applications. In the subsequent development of blockchain technology applications in smart cities, importance should be attached to the construction of blockchain underlying architecture and infrastructure, putting the building of blockchain basic service platforms in the first place, deep integration with technologies such as big data, artificial intelligence and the Internet of Things, and further improvement of regulatory and standard systems. What we want to solve the openness, exchange, integration, sharing and security of data resources that are isolated and segregated because of category, industry, sector and geography. Based on the analysis of blockchain technology in Part II III, as shown in Figure 4, we can divide the development of smart cities into four directions—people’s livelihood services, urban governance, industrial economy and ecological livability.

### 6.1. People’s Livelihood Services

**Figure 5:**
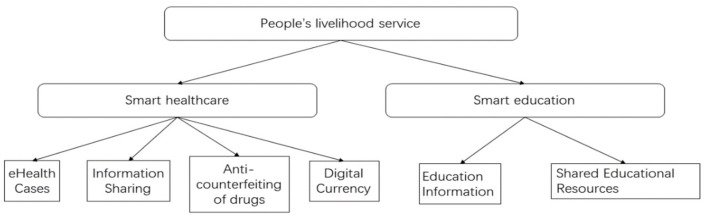
People’s livelihood service structure diagram.

The main application scenarios of blockchain technology in the field of livelihood services include: smart healthcare and smart education, as shown in Figure 5.

In recent years there have been numerous problems in the medical field and the masses have not been able to get better solutions to their medical problems. The use of blockchain technology can-to a certain extent, improve the current system construction in the medical field-enhance its efficiency and further promote the application of Internet+ medicine. As an important industry application scene, the medical field has seen a year-on-year increase in the proportion of major enterprises, government departments and investment institutions in China, and outside China making strategic investments in its layout, constantly accelerating the application of blockchain technology in the medical industry. Smart healthcare which includes electronic health cases, information sharing, anti-counterfeiting of drugs, and digital currency payments. We use technology to promote the development of medical informatization and ensure the storage and sharing of medical data, which includes all aspects of hospital and patient information. For example, policy data storage and sharing, medical and health records storage on the chain, etc.

**Figure 6:**
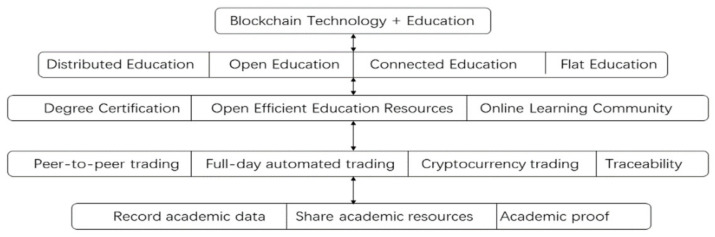
A new ecology of education based on blockchain technology.

Education has come a long way in the last 20 years, but there is still a long way to go before education is fully modernized. Technologies like blockchain can accelerate the process of modernizing education. Distributed ledgers, artificial intelligence, and electronic devices are slowly becoming the future direction of choice for educational tools. When blockchain is used in education, blockchain technology can enhance the transparency of education, such as submitting assignments and checking grades and learning progress, and it can improve the motivation of students to learn, and scholarships can be awarded using cryptocurrency. The main focus of smart education is to preserve information data of teachers, students and educational institutions, and to share resources, and to build efficient online learning communities through smart contracts, so that a series of tasks such as uploading, authenticating, flowing and sharing educational resources can be executed automatically, and reducing the cost of sharing resources, improving the efficiency of resource sharing and monitoring the community ecosystem in real time. The new ecosystem of ‘blockchain + education’ is formed based on the characteristics of blockchain technology such as efficiency and transparency, and it helps to innovate the education industry [92], as shown in Figure 6.

Blockchain technology can be used in education as a record for storing distributed learning; it can provide a trusted certificate system for online education; it can be used as a smart contract to complete educational contracts and depository; blockchain technology can be used as a copyright tool to mark academic achievements; and it can be a decentralized global knowledge base, and a knowledge currency [93]. In terms of practical applications, the MT Media Lab (MIT Media Lab) has released ‘Blockcerts’, a blockchain certificate project, an open standard for digital academic certificates based on the Bitcoin blockchain. ‘Blockcerts’ provides a decentralized authentication system. Because it relies on the most secure Bitcoin blockchain, its credentials are tamper-proof and verifiable. In addition, ‘Blockcerts’ can be used to issue any type of credentials, including professional certificates, transcripts, credits, or degrees [94]; the Holberton School, a software education institution in San Francisco, uses blockchain technology to record academic credentials for its schools and will start to share information on academic credentials on the blockchain starting in 2017. In addition to providing evidence of academic achievement, blocks can be used as a basis for measuring an individual’s intellectual wealth [95]. By analogy with Bitcoin, a block that records one’s academic achievements can also be used as a “knowledge currency”. In other words, the concept of “Learning is Earning” is used to promote education [96], and the above knowledge currency will also become a token (cryptocurrency) within the DAO organization, where students can earn cryptocurrency in the form of questions, answers, and posts, which will also motivate students to learn. This also proves the argument that ‘learning is earning’.

### 6.2. Urban Governance

**Figure 7:**
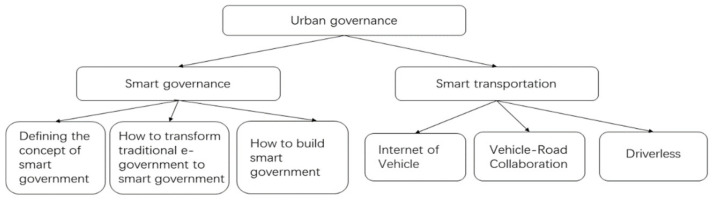
Urban governance structure diagram.

The key application scenarios of blockchain technology in the field of urban governance include: smart government, smart transportation, as shown in Figure 7.

So-called smart government is an e-government based on blockchain, cloud computing and other technologies. In these years, the development of e-government has encountered various problems that still need to be solved, such as low efficiency, data cannot be shared and so on. The first is to define the concept of e-government, which is understood by academics as the use of advanced information technology for collaborative governance of society and the provision of new and efficient services to the public to meet the changing social needs [97,98]. The second, how to develop from traditional government to e-government, which research shows can be developed in steps development and finally reach the level of e-government [99]. The third, how to build smart government specifically, some scholars have proposed a new government service system using GIS and cloud computing technology, which can visualize the operational information of the city [100]. This concept has been expanded, and some scholars have combined the concept of borderless with e-government to form, whose goal is to efficiently meet the needs of the public, means to integrate the process of government affairs across borders, and to collaborate organizations and information across borders weakening the organization of government functions. The goal is to efficiently meet the needs of the public by integrating government affairs processes across boundaries, collaborating organizations and information across boundaries, and weakening the organizational boundaries of government functions so that the entire government functions are truly integrated under the perspective of public services [101]. Borderless wisdom government is supported by blockchain technology, and the information of each department is stored in a distributed manner, and the mutual trust problem is solved between the nodes using consensus mechanisms, which finally forms a borderless government service system and improves the security and efficiency of government services.

Smart transportation, as one of the cores of smart cities, involves many integrated technologies such as Internet of Things, cloud computing, and big data, which enable the coordinated operation of people, vehicles, and roads [102]. With the characteristics of blockchain technology such as polycentricity, security and trustworthiness, and smart contracts, it can realize the construction of a more efficient transportation network, and solve the government, enterprise data sharing and Intelligent management of infrastructure and other issues. On the basis of ensuring open and transparent data circulation, data security is ensured to improve the efficiency of intelligent transportation operation. However, policy failure and control hijacking caused by information security have become major hidden dangers for the promotion and application of new technologies. The application of blockchain in Telematics technology can achieve more secure, reliable data storage and authentication through data encryption and consensus mechanisms. Providing sustainable information services and effective manner, ensuring data security to safeguard telematics technology security. At the same time, through the establishment of alliances and contracts, the effective and seamless integration of information collected by smart transportation field terminals, intelligent vehicle information, manual control commands, and road infrastructure information can be realized. Thus, effectively solving traffic congestion, parking difficulties, and other traffic hotspot problems [103].

**Figure 8:**
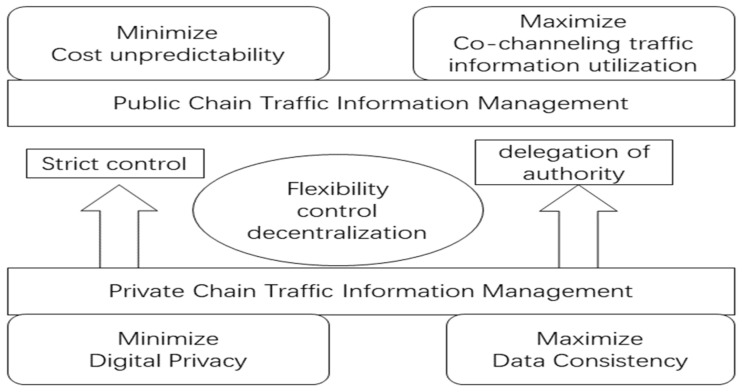
Blockchain collaborative traffic information management framework.

Take electric car charging piles as an example; at present, all countries in the world advocate reducing carbon emissions and using more clean energy, and the demand for electric cars is gradually increasing, but the number of charging piles is limited and the configuration is unbalanced. How to let users choose the right one for charging from the limited number of charging piles with uneven space distribution is an urgent problem to be solved. Users can log into the APP to check the distribution of available charging piles and then make a choice according to their own wishes. The whole transaction process is completed by a central processing entity in the background. Such an approach does not consider the variability of individual travelers’ needs. Therefore, by introducing the concept of blockchain and adopting a decentralized smart charging contract, we can effectively help users select the most convenient parking/charging location and choose the service completely independently. The specific architecture includes four layers: user layer, in-vehicle information interaction layer, smart contract layer, and target layer [104]. The figure below shows a new management model for new transportation, where multiple types of blockchains are managed collaboratively, as shown in Figure 8. For example, German energy giant Innogy and IoT platform company Slock.it have partnered to launch a blockchain-based peer-to-peer charging project for electric vehicles. Instead of signing any power supply contract with the power company, users can simply install the Share and Charge APP on their smartphones and complete user verification to charge at Innogy’s charging posts across Europe, with tariffs automatically determined by a backend program in real-time based on the prevailing and local grid load. Thanks to blockchain technology, the entire charging and tariff optimization process is fully traceable and searchable, thus significantly reducing trust costs. When charging is needed, an available charging station nearby is found from the app and payment is made according to the price in the smart contract. However, this type of charging is not yet popular, even in Germany, where Ethereum wallets are only an option for some people, but it will still accelerate the connection between cryptocurrencies and real life

### 6.3. Industrial Economy

**Figure 9:**
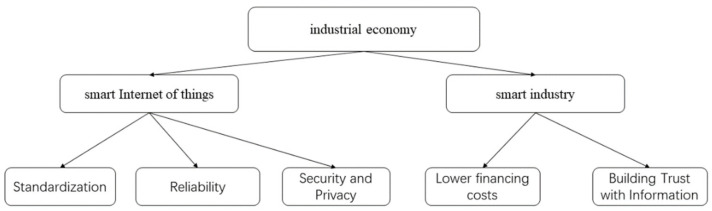
Industrial economy structure diagram.

The key application scenarios of blockchain technology in the industrial economy include: intelligent Internet of things, intelligent industry, as shown in Figure 9.

As an emerging technology, IoT has gradually penetrated into every aspect of life. The security of blockchain technology makes IoT + blockchain be more and more attention. Using blockchain technology can solve the original problems of IoT such as no standardization, information security. Internet of things is an Internet-based and can make all the ordinary physical objects connected to the information carrier. After the development of wired and wireless networks, especially to the 5G era, it can realize the network interconnection of people and people, people and things, things and things. With the development of artificial intelligence, big data, cloud computing and other technologies to promote the development of the Internet of things, in the future, the Internet of things still need to pay attention to the standardization issue. About the Internet of things, there is no standardized construction policy, there is no unified communication protocol, communication interface, etc., there is the same protocol to make the device interconnection, and each company wants to implement their own communication protocol, in order to be the formation of industry development barriers, but now is still in the early stage of industry development. Additionally, the reliability issue. As the current IoT architecture is to aggregate all data into a central control system, all the data have the risk of being controlled and modified at will, and in the process of data transmission may go through multiple links, the authenticity and integrity of the data cannot be guaranteed, and then, security and privacy issues. In the field of IoT, the centralized service architecture stores and forwards all monitoring data and signals through a central server, and a large amount of user data information is stored in the central server. Although IoT operators keep emphasizing that they can effectively protect user data and privacy, security breaches and privacy leaks still occur, making a large number of users unable to trust that their privacy is secure [105].

**Figure 10:**
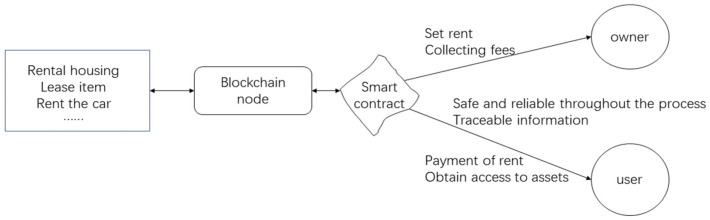
New sharing economy structure diagram.

During the long-term development and evolution of IoT, the following 9 industry pain points have been encountered: device security, personal privacy, architectural rigidity, communication compatibility and multi-subject collaboration 5 major pain points. The improvements of blockchain technology for IoT are: cost reduction, privacy protection, identity identification, traceability, and cross-subject collaboration. The current development status is: leading IoT companies introduce blockchain technology in large numbers, for example, IBM launched blockchain services for Bluemix cloud platform back in 2016, Amazon chose to start cooperation with DCG, a digital currency company; from the perspective of traditional power companies, they mainly invest in different pilot projects by cooperating with startups, setting up subsidiaries, or even buying startups to create distributed energy systems and peer-to-peer energy trading platforms. Including Sweden’s state-run power company VattenFall (Waterfall Power), which invested in a startup (PowerPeers) in Amsterdam, the Netherlands, and to build an energy-sharing platform that allows consumers to freely choose their power channels, and Germany’s Rheinland (RWE), which partnered with startup Slock.it to launch ‘s BlockCharge EV charging project, these platforms have terminal payment systems that support the use of cryptocurrencies, which is also driving the development of cryptocurrencies in practical applications [106]. The main application scenarios: sensor data deposition and traceability; new sharing economy; energy trading; charging cars and charging piles, communication and intelligence for drones. In the following, the new ‘sharing’ economy is used as an example, as shown in Figure 10. The whole blockchain network is built based on the blockchain. Based on the smart contract system, the asset owner sets the rent, deposit and related rules to complete the binding of various ‘locks’ with the asset, and the end of user pays the corresponding rent and deposit to the asset owner through the APP to obtain the key and then obtain the right to use the asset. At the end of the use, return the item and get back the deposit, the payment system here will try to use cryptocurrency.

Smart industry is mainly involved in the supply chain finance with the back ground of blockchain, using blockchain as a way of information transfer and data taking, which can effectively reduce the cost of trust and loan taking. reducing the cost of enterprise financing, focusing on solve the problems of financing difficulties and high capital costs of upstream and downstream SMEs [107]. The project supply chain information platform is optimized by combining blockchain technology in engineering projects, and integrated with project management function information integration and project information collection system to build an engineering project information integration management platform based on smart industry [108]. They are also involved in industrial equipment identity management, equipment access control, equipment registration management, and equipment operation status supervision, etc. The execution of blockchain smart contracts is used to obtain and verify equipment identity, and blockchain technology can guarantee the security of relevant data and ensure that the enterprises of the industrial chain can access credible and consistent equipment operation data. The distributed storage, tamper-evident and encryption algorithms of blockchain technology can be used to realize data exchange among various entities of the industrial Internet, and the production and manufacturing data of each enterprise can be stored on the blockchain, while realizing the sharing of data of other enterprises.

### 6.4. Ecological Livability

**Figure 11:**
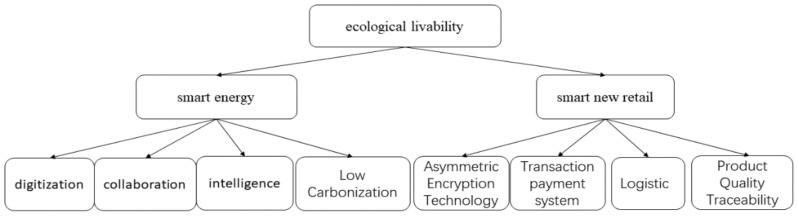
Ecological livability structure diagram.

The key application scenarios of blockchain technology in the field of ecological livability include: smart energy, smart new retail, as shown in Figure 11.

With the development of industrialization in China, energy, as the support of national life, has received more and more attention in terms of security, efficiency and trust in its development and transaction process. Combining blockchain technology with energy can optimize the traditional energy transaction mode, improve transaction efficiency, and promote the healthy development of energy [109]. Some scholars have elaborated the current situation and challenges faced by blockchain technology in the application of integrated energy system, analyzed the key issues to be solved in the construction of energy blockchain system, and made an outlook on the future development of integrated energy based on blockchain [110], and some scholars have also conducted research on the optimization of energy costs [111,112]. However, there are still many challenges when applying blockchain technology. Firstly, the energy interaction information of the energy system is dynamically changing, and the data throughput is significantly more than the blockchain application scenario of transaction settlement, which causes difficulties for the efficient operation of the system, and even the communication delay and information blockage. Secondly, the consensus mechanism is wasteful of resources for the high energy demand, and it is needed to reduce the energy consumption in the future. The data of the energy production chain is generated and stored locally by each energy vendor, and the data of each energy vendor is disconnected and unconnected. It can be combined with IoT devices to realize safe storage and sharing of production data by applying blockchain technology based on data collection, improving monitoring accuracy, mining data value, and creating an information basis for government supervision.

**Figure 12:**
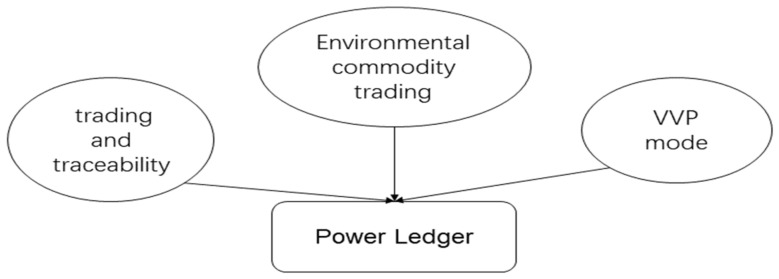
Power ledger module.

There are already a large number of use cases in real-world applications, for ex ample, the US energy company LO3 Energy partnered with Siemens Digital Grid in April 2016, and the Bitcoin development company Consensus Systems to create the Brooklyn Microgrid-an interactive grid platform based on the blockchain system the project is the world’s first energy marketplace based on blockchain technology. This microgrid project enables peer-to-peer electricity trading for residents between communities, allowing users to access data related to electricity generation and consumption in real time through smart meters and buy or sell electricity and energy to others through the blockchain; Power Ledger was founded in Perth, Australia, by Ledger Assets, an Australian blockchain software company. Power Ledger uses blockchain-based software to build a P2P system for trading surplus solar power to the grid. Unlike the PoW (proof of work) mechanism used by Bitcoin, Power Ledger uses a POS (proof of stake) mechanism, and the blockchain was developed by Ledger Assets and is called Ecochain [113]. The above cases are representative cases, where the Power Ledger platform has modular and scalable features, mainly through three aspects of sustainable energy aspects of business, and any individual module can be extended on demand, namely energy trading and traceability, VVP model, and environmental commodity trading [114], as shown in Figure 12.

The energy trading and traceability product is called xGrid. xGrid allows trading renewable energy across the grid or behind the meter, improving the relationship between customers, retailers, and the distribution network to make it more efficient. Specifically, xGrid supports customers in selling energy from solar panels to other energy consumers connected to the same grid. From generation to consumption, it manages the settlement of energy transactions between the two parties, allowing dynamic price discovery at intervals as short as five minutes. Users can customize their profiles to sell power in their preferred way, and the VPP model software will detect when wholesale market prices are peaking. Managing stored power, helping customers maximize their return on investment while supporting the grid with clean solar energy. The environmental commodity trading product, called TraceX, is a digital marketplace for trading and settling environmental commodities, such as Renewable Energy Certificates (RECs) and Time-based Environmental Certificates (T-EACs).

With the increasing maturity of blockchain technology, this has brought new opportunities for the development of various fields in China. The main problems faced in China’s new retail supply chain are also found, namely, transaction payment security, logistics information and commodity traceability. It was shown that the asymmetric encryption technology in blockchain technology achieves the upgrading and optimization of the retail supply chain, while blockchain technology makes it possible to realize the decentralization of transaction payments, thus improving the effectiveness of logistics information and the integrity of commodity traceability [115]. Using the distributed bookkeeping and non-tampering characteristics of blockchain, the supply chain data of commodities are stored in a distributed manner, access rights and encryption verification are set, the data exchange, and sharing in the supply chain process, and the business operation process simplified to realize the overall efficiency improvement and optimization of the supply chain. Using blockchain technology to build a digital supply chain, especially the supply chain management in cross-border trade, the distributed bookkeeping, and tamper-evident characteristics of blockchain are used to distribute the supply chain data of commodities for storage, authorized access and encryption verification, simplifying the data exchange and sharing in the supply chain process and the business operation process. Realizing the overall efficiency improvement and optimization of the supply chain.

The above four aspects do not cover all of the smart city, but can give a direction to the blockchain-based urban governance. It can be seen that when the industry is combined with blockchain it can bring security and efficiency improvement, moreover, it can reduce the cost of governance.

## 7. Conclusions

This study analyses the development direction of a smart city based on blockchain technology and cryptocurrency and uses a literature review to compare the six areas of blockchain in China and other countries. We included the hot spots and research preferences of the application layer, contract layer, consensus layer, incentive layer, network layer and data layer of the blockchain, and found that China’s research is more preference application of landing and industry, while international research is more preferred to the research of the underlying technology of finance and blockchain. Based on the analysis of cryptocurrencies and smart city, cryptocurrencies will get more market support in the future, and currently ‘metaverse’, ‘DAO’, ‘NFT’, ‘Web 3.0’ are developing rapidly, cryptocurrencies may be combined with smart cities to reshape the whole financial industry and network world. This paper introduces the concept of Kolmogorov entropy to cryptocurrencies and smart cities to measure the level of disorder within the system. This study is a qualitative study, and we will do more quantitative studies in the future to measure specific monetary entropy and Kolmogorov entropy, crypto-economic entropy, urban entropy, etc. The study may help scholars interested in blockchain to learn the basic knowledge of blockchain, and at the same time helps to understand the global hotspots of entropy and the research direction of entropy.

## Figures and Tables

**Table 1:** The factors of influencing the entropy values.

	Traditional Monetary Market	Cryptocurrency Market
The factors of influencing the entropy values	Inflation rate	NFT
	Fiscal deficit level	DAO
	Interest rate volatility	Web 3.0
	The cost of currency management	Metaverse

**Table 2:** Different science and technology concerns for each layer at China and outside China.

Blockchain Architecture		Research in China	Research Outside China
Application layer	Programmable currency	Tokenized Open Finance [8]	Investment [9]
		Trading account traceability [10]	Payment [11,12]
			Anti-money Laundering [13]
			Identity [14]
	Programmable finance	Trading System [15]	Business Economy [16]
		E-commerce platform [17,18,19]	Finance [20,21]
		Sharing Economy [22]	
	Programmable society	Supply Chain [23]	Social Governance [24,25]
		Social Governance [26,27,28,29,30,31,32,33,34]	Identity management [35]
		Education [36,37,38,39,40]	Business Process [41]
		Taxation [42,43,44,45] Medical [46] Rights Protection [47] Intellectual Property [48,49]	
Contract layer	Smart Algorithm	State Machine Network [50] Domain State Machine Vector clock state machine	Business process modelling notation [51] Reparo Protocal [52]
	Smart Contract	Double auction pricing contract	E-Bidding [53]
	Smart Scripts	Smart scripts incorporating machine learning [54]	Picture Hash [55]
Incentive layer	Game model	Reputation model based on multiple games	Reputation incentive model [56]
	Incentives	Stochastic equity proof mechanism [57]	Authoritative participation and internal drive incentive mechanism [58]
Consensus layer	PoS DPoS	Multi-group proxy consensus mechanismMG-DPoS	Improving consensus in proof of stake protocols [59]
	PoW	estPoW	Proof-of-Trust (PoT) [60]
		Zero-determinant strategy based on game theory [61]	Randomly Elected Blockchain (REBC) [62]
	PBFT	Dynamic consensus mechanism	Open Business Environment-PBFT [63]
Network layer	P2P Network	Network layer node configuration trading subsystem [64]	IoT trust management [65]
	Spread mechanism	Blockchain network protocol based on IPv6 [66]	Dual-Channel Parallel Broadcast model (DCPB) [67]
		Trusted service quality model [68]	
	Authentication mechanism	SDN control layer security mechanism construction method [69]	Network is under the influence of such attacks [70]
Data layer	Data Block Asymmetric encryption	BigchainDB [71]	Health data based on an extension of permissioned blockchain [72]
		Block chain data safe transmission method based on SCTP protocol [73]	Graphic data encryption solution based on private blockchain
	Chain Structure	Two-layer blockchain user trust negotiation model [74]	Complex networks modelling framework [75]
	Timestamp Hash Merkle Tree	Attack detection model based on Merkle hash tree structure [76]	Data Structure of Streaming Authentication Based on Merkle Tree [77]

**Table 3:** Differences Between China and International Research using blockchain technology.

Blockchain Technology	Differences between Chinese and International Research	
	In China	Outside China
Application Layer	Focused on the real economy	Focused on the virtual economy
Contract Layer	Linked to the industrial chain	Blockchain contract protocol improvements
Actuator Layer	Reputation-based game model design	
Consensus Layer	Improved consensus algorithm based on existing PoW, PoS, DPoS, PBFT	
Network Layer	Improvements through external software and services	Improvements of the network layer itself
Data Layer	Application to Security	Improvements of the data layer itself

**Table 4:** DAO Landscape around world.

DAO Landscape	Examples
DAOoperating systems	DAOStack, colony, Orca etc.
Investments DAOs	theLao, BitDao, Metacartel etc.
Grants DAO	Uniswap Grants, Aave Grants etc.
Collector DAOs	PleasrDao, herstoryDAO etc.
protocolDAOs	Curve, AAVE, Sushi etc.
SerivceDAOs	partyDAO, metaverseDAO etc.
SocialDAOs	FWB, seedclub etc.
Media DAOs	GCR etc.

## Data Availability

Not applicable.

## References

[1] Jakimowicz A. (2020). The Role of Entropy in the Development of Economics. Entropy.

[2] Grassberger P., Procaccia I. (1983). Estimation of the Kolmogorov entropy from a chaotic signal. Phys. Rev. A.

[3] Gunay S., Kaşkaloğlu K. (2019). Seeking a Chaotic Order in the Cryptocurrency Market. Math. Comput. Appl..

[4] Fistola R., La Rocca R.A. (2014). The Sustainable City and the Smart City: Measuring urban entropy first. WIT Trans. Ecol. Environ..

[5] Dehouche N. (2021). Revisiting the Volatility of Bitcoin with Approximate Entropy. Cogent Econ. Financ..

[6] Pele D.T., Mazurencu M. (2019). Using High-Frequency Entropy to Forecast Bitcoin’s Daily Value at Risk. Entropy.

[7] Santoro D., Grilli L. (2020). A Statistical Ensemble Based Approach for Entropy in Cryptocurrencies Markets. 13th Chaotic Modeling and Simulation International Conference.

[8] Wu T. (2020). Advantages, constraints and promotion strategies of open finance based on blockchain. Econ. Asp..

[9] Zhu L., Gao F., Shen M., Li Y., Zheng B., Mao H., Wu Z. (2017). Summary of research on blockchain privacy protection% Survey on Privacy Preserving Techniques for Blockchain Technology. Comput. Res. Dev..

[10] Wan G., Sun T. (2017). Research on the Ideal, Reality and Regulatory Countermeasures of “Blockchain + Securities”. Shanghai Financ..

[11] Pu D., Fan C., Liang H. (2018). Construction and application of e-commerce platform system based on blockchain perspective. China Circ. Econ..

[12] Zhou M., Qin W. (2019). Reconstruction of e-commerce credit system in the era of blockchain 3.0. Learn. Pract..

[13] Li D. (2020). Research on the Coupling Development of Digital Economy, Cross-border E-commerce and Digital Trade—Also on the Application of Blockchain Technology in the Three. Theor. Discuss.

[14] Yan Z. (2020). A new sharing economy model based on blockchain technology. Soc. Sci. Res..

[15] Zhang X. (2018). Optimization of supply chain management model based on blockchain. China Circ. Econ..

[16] Zhang X., Zhang H., Guo X., Wen Z. (2017). Research and analysis of electronic voting and election system based on blockchain. Electron. Technol. Appl..

[17] Lu M. (2020). Research on the Reconstruction of Blockchain Technology and Social Credit System. Lanzhou Acad. J..

[18] Shi D. (2019). On the value and risk of blockchain technology for digital copyright governance. Sci. Technol..

[19] Yan Z., Liu L., Li Q. (2018). Cooperative Evolution of Parallel Society under Blockchain System. China Sci. Technol. Forum.

[20] Liu Y., Zhang Y., Wu Y., Zheng C. (2020). Blockchain technology and document archive management: Two-way thinking of technology and management. Arch. Newsl..

[21] Lin M., Zhang Z. (2019). “Blockchain + Production” Promotes Enterprise Green Production—A New Thinking on the Hand of Government. Econ. Trends.

[22] Zhao J., Meng T. (2019). Technology Empowerment: How Blockchain Reshapes Governance Structure and Model. Contemp. World Soc..

[23] Gao Q., Zhang J. (2019). Blockchain and Transformation of Global Economic Governance—Based on the Perspective of Con-structing a Global Justice Economic Order. Academia.

[24] Yang H. (2018). The new globalization process after the volatility and the new issues of global governance—Transnational coupling of blockchain, human mobility and authority. Acad. Circ..

[25] Jin Y. (2021). Demand analysis and technical framework of blockchain + education. China Audio-Vis. Educ..

[26] Xu J., Wang J. (2017). Trust Building of Academic Publishing Based on Blockchain Technology. Publ. Sci..

[27] Xu T. (2017). Application and Challenge of Blockchain Technology in Education and Teaching. Mod. Educ. Technol..

[28] Li Z., Qiu T., Li K. (2019). Research on Credit Banking System Based on Blockchain Technology. Mod. Educ. Technol..

[29] Chen X. (2019). Blockchain technology and its application in audit experiment teaching. China Audio-Vis. Educ..

[30] Yang Y., Du J., Luo X. (2019). Analysis of the impact of blockchain technology on both parties of tax collection. Tax Res..

[31] Ren C. (2018). Research on tax collection and management model based on blockchain technology. Tax Res..

[32] Li W., Liu H., Deng X. (2018). Application of Blockchain Technology to Promote Credit Management of Tax Payment in my country. Tax Res..

[33] Tang X., Zhou H. (2018). Construction of tax governance framework based on blockchain technology. Tax Res..

[34] Dong D., Wang X. (2019). Research on electronic medical record sharing based on blockchain. Comput. Technol. Dev..

[35] Zhu J. (2018). Blockchain finance consumer rights protection: The path of experimental regulation. Nanjing Soc. Sci..

[36] Ding X. (2018). Thoughts on the integration of blockchain into copyright insurance. Nanjing Soc. Sci..

[37] Zhang L., Zhou Y., Xue L. (2018). Intellectual Property Management and Policy Research of Strategic Emerging Industries Based on Blockchain Technology. China Sci. Technol. Forum.

[38] Huang B., Deng X., Yu Z., Wang C., Guo D., Yang Z. (2018). A Data Synchronization Method Based on the Operation of Block-Chain State Unit Network. China Patent.

[39] Zhang Y., Wang Y., Yang Z., Yang R. (2019). Blockchain smart contract script design based on machine learning. Inf. Eng..

[40] Hao S., Xu W., Tang Z. (2018). Research on scientific data sharing blockchain model and implementation mechanism. Inf. Theory Pract..

[41] Tang C., Yang Z., Zheng Z., Cheng Z.Y. (2017). Analysis and Optimization of Game Dilemma in PoW Consensus Algorithm. J. Autom..

[42] Zhu X. (2018). Blockchain-Based Transaction Buffer/Acceleration Method and Blockchain Transaction Processing System. China patent.

[43] Lin J. (2019). Promote the development of blockchain technology through the integration of IPv6 technology. Comput. Prod. Circ..

[44] Wang L., Zhao X. (2019). Research on service composition strategy in cloud computing environment based on blockchain mechanism. J. Comput. Appl..

[45] Weng J., Weng J., Liu J., Wei K., Luo W. (2017). Blockchain-Based Software-Defined Network Control Layer Security Mechanism Construction Method. China Patent.

[46] Jiao T., Nie T., Shen D., Li X. (2019). Blockchain database: A queryable and tamper-proof database. J. Softw..

[47] Deng E. (2017). A Method for Secure Transmission of Blockchain Data Based on SCTP Protocol. China Patent.

[48] Yang M., Zhang S., Zhang H., Liu N., Gan B. (2019). User trust negotiation model based on two-layer blockchain in heterogeneous alliance system. J. Appl. Sci..

[49] Wang M., Duan M. (2018). Merkle Hash Tree Structure Based Blockchain Second Preimage Attack. Inf. Netw. Secur..

[50] Andriole S.J. (2020). Blockchain, Cryptocurrency, and Cybersecurity. IT Prof..

[51] Rezaeibagha F., Mu Y. (2019). Efficient Micropayment of Cryptocurrency from Blockchains. Comput. J..

[52] Zhong L., Wu Q., Xie J., Guan Z., Qin B. (2019). A secure large-scale instant payment system based on blockchain. Comput. Secur..

[53] Yin H.H.S., Langenheldt K., Harlev M., Mukkamala R.R., Vatrapu R. (2019). Regulating Cryptocurrencies: A Supervised Machine Learning Approach to De-Anonymizing the Bitcoin Blockchain. J. Manag. Inf. Syst..

[54] Juhasz P.L., Steger J., Kondor D., Vattay G. (2018). A Bayesian approach to identify Bitcoin users. PLoS ONE.

[55] Ante L. (2020). A place next to Satoshi: Foundations of blockchain and cryptocurrency research in business and economics. Scien-tometrics.

[56] Eyal I. (2017). Blockchain Technology: Transforming Libertarian Cryptocurrency Dreams to Finance and Banking Realities. Computer.

[57] Hegadekatti K., Yatish S.G. (2017). The Programmable Economy: Envisaging an Entire Planned Economic System as a Single Computer through Blockchain Networks. MPRA Pap..

[58] Aste T., Tasca P., Matteo T.D. (2017). Blockchain Technologies: The Foreseeable Impact on Society and Industry. Computer.

[59] Campbell-Verduyn M. (2019). Introduction to special section on blockchains and financial globalization. Glob. Netw..

[60] Abe R., Watanabe H., Ohashi S., Fujimura S., Nakadaira A. Storage Protocol for Securing Blockchain Transparency. Proceedings of the 2018 IEEE 42nd Annual Computer Software and Applications Conference (COMPSAC).

[61] Wang S., Ouyang L., Yuan Y., Ni X., Han X., Wang F.Y. (2019). Blockchain-Enabled Smart Contracts: Architecture, Applications, and Future Trends. IEEE Trans. Syst. Man Cybern. Syst..

[62] Thyagarajan S.A.K., Bhat A., Magri B., Tschudi D., Kate A. (2020). Reparo: Publicly Verifiable Layer to Repair Blockchains. arXiv.

[63] Manimaran P., Dhanalakshmi R. Blockchain-Based Smart Contract for E-Bidding System. Proceedings of the International Conference on Intelligent Communication & Computational Techniques.

[64] Lamberti F., Gatteschi V., Demartini C., Pelissier M., Gomez A., Santamaria V. (2018). Blockchains Can Work for Car Insurance: Using Smart Contracts and Sensors to Provide On-Demand Coverage. IEEE Consum. Electron. Mag..

[65] Wang E.K., Liang Z., Chen C.M., Kumari S., Khan M.K. (2020). PoRX: A reputation incentive scheme for blockchain consensus of IIoT. Future Gener. Comput. Syst..

[66] Okada H., Yamasaki S., Bracamonte V. Proposed classification of blockchains based on authority and incentive dimensions. Proceedings of the International Conference on Advanced Communication Technology.

[67] Wu W., Gao Z. (2020). An Improved Blockchain Consensus Mechanism Based on Open Business Environment. IOP Conf. Ser. Earth Environ. Sci..

[68] Zou J., Ye B., Qu L., Wang Y., Orgun M.A., Li L. (2018). A Proof-of-Trust Consensus Protocol for Enhancing Accountability in Crowdsourcing Services. IEEE Trans. Serv. Comput..

[69] Feng L., Hui Z., Tsai W.T., Sun S. (2019). System architecture for high-performance permissioned blockchains. Front. Comput. Sci..

[70] Leonardos S., Reijsbergen D., Piliouras G. Weighted Voting on the Blockchain: Improving Consensus in Proof of Stake Protocols. Proceedings of the IEEE International Conference on Blockchain and Cryptocurrency (ICBC 2019).

[71] Yang D., Jeon S., Doh I., Chae K. Randomly Elected Blockchain System based on Grouping Verifiers for Efficiency and Security. Proceedings of the 2020 22nd International Conference on Advanced Communication Technology (ICACT).

[72] Chicarino V., Albuquerque C., Jesus E., Rocha A. (2020). On the detection of selfish mining and stalker attacks in blockchain networks. Ann. Telecommun..

[73] Frahat R.T., Monowar M.M., Buhari S.M. Secure and Scalable Trust Management Model for IoT P2P Network. Proceedings of the 2019 2nd International Conference on Computer Applications & Information Security (ICCAIS).

[74] Ito K., Tago K., Jin Q. i-Blockchain: A Blockchain-Empowered Individual-Centric Framework for Privacy-Preserved Use of Personal Health Data. Proceedings of the 2018 9th International Conference on Information Technology in Medicine and Education (ITME).

[75] Ferretti S., D’Angelo G. (2020). On the Ethereum Blockchain Structure: A Complex Networks Theory Perspective. Concurr. Comput. Pract. Exp..

[76] Liu F., Yang C., Yu X., Qi J. (2022). Spectrograph Convolutional Neural Network for Decentralized Double Differential Privacy. Inf. Network Secur..

[77] Liu F., Zhang J., Zhou J., Li M., Kong D., Yang J., Qi J., Zhou A. (2022). Blockchain Cross-Chain Asset Interaction Protocol Based on Improved Hash Time Lock. Comput. Sci..

[78] Xu J., Wei L., Zhang Y., Wang A., Zhou F., Gao C.Z. (2018). Dynamic Fully Homomorphic encryption-based Merkle Tree for lightweight streaming authenticated data structures. J. Netw. Comput. Appl..

[79] Waqas P., Byun Y. (2020). A Blockchain-Based Secure Image Encryption Scheme for the Industrial Internet of Things. Entropy.

[80] Lv T., Liu F. (2021). Research on computing power network under the background of digital economy. J. Beijing Jiaotong Univ. (Soc. Sci. Ed.).

[81] Liu F., Wang Y., Yang J., Zhou A., Qi J. (2021). A blockchain-based high-threshold signature protocol integrating DKG and BLS. Comput. Sci..

[82] Liu F., Yang J., Qi J. (2021). Blockchain Two-Party Elliptic Curve Based on Hash Proof System. Inf. Netw. Secur..

[83] Liu F., Yang J., Li Z., Qi J. (2020). Research on an Endogenous Data Security Interaction Protocol for Dual-Central-Two-chain Architecture. J. East China Norm. Univ. (Nat. Sci. Ed.).

[84] Dyhrberg A.H. (2016). Bitcoin, gold and the dollar—A GARCH volatility analysis. Financ. Res. Lett..

[85] Huang Y., Duan K., Mishra T. (2021). Is Bitcoin really more than a diversifier? A pre- and post-COVID-19 analysis. Financ. Res. Lett..

[86] Mariana C.D., Ekaputra I.A., Husodo Z.A. (2021). Are Bitcoin and Ethereum safe-havens for stocks during the COVID-19 pandemic?. Financ. Res. Lett..

[87] Disli M., Nagayev R., Salim K., Rizkiah S.K., Aysan A.F. (2021). In search of safe haven assets during COVID-19 pandemic: An empirical analysis of different investor types. Res. Int. Bus. Financ..

[88] Kwapień J., Wątorek M., Drożdż S. (2021). Cryptocurrency Market Consolidation in 2020–2021. Entropy.

[89] “The Transaction Volume of NFT” [EB/OL]. https://nonfungible.com/market/history.

[90] Wang Q., Li R., Wang Q., Chen S. (2021). Non-Fungible Token (NFT): Overview, Evaluation, Opportunities and Challenges. arXiv.

[91] Mnif E., Jarboui A., Mouakhar K. (2020). How the cryptocurrency market has performed during COVID 19? A multifractal analysis. Finance Research Letters.

[92] Nakamoto S. (2008). Bitcoin: A peer-to-peer electronic cash system. Decentralized Bus. Rev..

[93] Li Q., Zhang X. (2017). Blockchain:Promoting openness and credibility of education with technology. J. Distance Educ..

[94] Schmidt P. ‘Blockcerts’: Open Standard for Blockchain Certificates Created by MIT[EB/OL]. https/www.8btc.com/article/107456.

[95] Sharples M., Domingue J. (2016). The Blockchain and Kudos: A Distributed System for Educational Record, Reputation and Reward. European Conference on Technology Enhanced Learning.

[96] Wang A. Blockchain Technology and Its Applications [EB/OL]. https://opensiuc.lib.siu.edu/cgi/viewcontent.cgi?article=1020&context=asars.

[97] Fei J., Jia H. (2015). The path selection of public service platform provided by government APP from the perspective of smart government. E-Government.

[98] Yu S., Yang D., Wang J., Zhang Y., Wang J. (2013). Big data-based smart government portal: From concept to practice. E-Government.

[99] Luo X., Yu B., Yao M. (2014). Analysis of e-government development stages from the perspective of information chain. Libr. Stud..

[100] Lv Z.H., Li X.M., Wang W.X., Zhang B., Hu J., Feng S. (2018). Government affairs service platform for smart city. Future Gener. Comput. Syst. Int. J. ESci..

[101] Hu M., Ma J. (2019). Research on the mechanism of promoting borderless smart government in the perspective of information collaboration. Intell. Data Work..

[102] Yuan Y.K., Zhang Y., Wei T. (2015). Review of key technologies and applications of intelligent transportation. Electron. Technol. Appl..

[103] Zhang H.-L. (2020). Application scenarios and challenges of blockchain technology in the transportation field. China Transp. Informatiz..

[104] Wang M., Gong Z. ‘Converging Applications of Blockchain Technology in the Field of Intelligent Transportation’ [EB/OL]. https://www.sohu.com/a/237977066_649849.

[105] Feng Y., Wang F. (2021). Blockchain-based application of Internet of things technology. Internet Things Technol..

[106] ‘Blockchain in the Internet of Things’ [EB/OL]. https://blog.csdn.net//bigtree_3721/article/details/79517416.

[107] Na L. (2020). New generation of information technology to enable intelligent development of coal industry. Coal Eng..

[108] Yang D.Q., Yue A., Yang R. (2019). Research on integrated management of engineering project information under smart construction-application based on blockchain technology. Constr. Econ..

[109] Yu S.B., Zheng D.D. (2020). Application and prospect of blockchain technology in the field of energy and power. Huadian Technol..

[110] Zhang Y., Wang L., Wu J., Yuan R., Li M. (2020). Blockchain and integrated energy systems: Applications and perspectives. China Sci. Found..

[111] Huang X., Zhang Y., Li D., Han L. (2019). An optimal scheduling algorithm for hybrid EV charging scenario using consortium blockchains. Future Gener. Comput. Syst..

[112] Andoni M., Robu V., Flynn D., Abram S., Geach D., Jenkins D., McCallum P., Peacoack A. (2019). Blockchain technology in the energy sector:A systematic review of challenges and opportunities. Renew. Sustain. Energy Rev..

[113] ‘Trends and Cutting-Edge Applications of Energy Blockchain, IoT Technologies in Smart Grid’ [EB/OL]. https://www.cnblogs.com/newstart/p/10594471.html.

[114] ‘Why Does Power Ledger Favor Solana?’ [EB/OL]. https://weibo.com//ttarticle/p/show?id=2309404668673431306451&sudaref=www.baidu.com.

[115] Wang Q., Chen Z., Wang P. (2020). Application of blockchain technology in new retail supply chain. Bus. Econ. Res..

